# Co-morbid disorders and sexual risk behavior in Nigerian adolescents with bipolar disorder

**DOI:** 10.1186/1755-7682-2-16

**Published:** 2009-06-01

**Authors:** Muideen O Bakare, Ahamefule O Agomoh, Peter O Ebigbo, Gabriel M Onyeama, Julian Eaton, Jojo U Onwukwe, Kevin O Okonkwo

**Affiliations:** 1 Child and Adolescent Unit, Federal Neuro-Psychiatric Hospital, New Haven, Enugu, Enugu State, Nigeria; 2 General/Forensic Unit, Federal Neuro-Psychiatric Hospital, New Haven, Enugu, Enugu State, Nigeria; 3 Department of Psychological Medicine, University of Nigeria Teaching Hospital, (UNTH), Enugu, Enugu State, Nigeria; 4 West Africa CBM National Co-ordination Office, PO Box 8451, Wuse, Abuja, Nigeria; 5 Community Psychiatry Unit, Federal Neuro-Psychiatric Hospital, New Haven, Enugu, Enugu State, Nigeria

## Abstract

**Background:**

Adolescent onset bipolar disorder often presents with co-morbid disorders of which psychoactive substance use disorders are notable. Mania symptoms and co-morbid psychoactive substance use disorders prone adolescents with bipolar disorder to impulsivity, impaired judgment, and risk taking behavior which often includes sexual risk behavior. There are dearth of information on pattern of co-morbid disorders and sexual risk behavior in adolescent onset bipolar disorder in Nigeria. This study assessed the prevalence and pattern of co-morbid disorders and determined associated factors of sexual risk behavior among adolescents with bipolar disorder.

**Methods:**

Socio-demographic information was obtained from the adolescents using socio-demographic questionnaire. Clinical interview, physical examination and laboratory investigations were employed to establish co-morbid disorders in these adolescents during the outpatient follow up visits over a one year period.

**Results:**

A total of forty six (46) adolescents with bipolar disorder were followed up over a one year period. Twenty two (47.8%) of the adolescents had co-morbid disorders with cannabis use disorders, alcohol use disorders, conduct disorder with or without other psychoactive substance use accounting for 23.9%, 8.7%, 13.0% respectively and HIV infection, though a chance finding accounting for 2.2%. Twenty one (45.7%) of the adolescents had positive history of sexual risk behavior, which was significantly associated with presence of co-morbid disorders (p = 0.003), level of religion activities in the adolescents (p = 0.000), and marital status of the parents (p = 0.021).

**Conclusion:**

When planning interventions for children and adolescents with bipolar disorder, special attention may need to be focused on group of adolescents with co-morbid disorders and propensity towards impulsivity and sexual risk behavior. This may help in improving long term outcome in this group of adolescents.

## Background

Co-morbid conditions had been known to characterize adolescent onset bipolar disorders [1-3] and co-morbid disorders impact negatively on outcome in adolescents with bipolar disorder [1,2,4,5]. Youths with bipolar disorder had been found to be prone to sexual abuse [6] and often exhibit sexual risk behavior [7]. Family and social dynamics had also been shown to influence co-morbidity and outcome in adolescent onset bipolar disorder [5].

There are dearth of information on prevalence and pattern of co-morbid disorders in adolescent onset bipolar disorder in Nigeria and other sub-Saharan African countries. Information is also limited on sexual risk behavior and its associated factors in adolescent onset bipolar disorder in this environment.

Attempt was made by this study to determine the prevalence and pattern of co-morbid diagnoses in Nigerian adolescents with bipolar disorder. It also determined the prevalence of sexual risk behavior and its associated factors in these adolescents. It assessed the association between co-morbid disorders and history of sexual risk behavior among the adolescents.

## Methods

### Location

Location of the study was the outpatient unit of Federal Neuro-Psychiatric Hospital, New Haven, Enugu (FNHE), Nigeria.

### Participants

The participants are adolescents attending the outpatient unit of FNHE, Nigeria on outpatient follow up visits with diagnosis of either bipolar I or bipolar II disorder based on Diagnostic and Statistical Manual of Mental Disorders, fourth edition (DSM-IV) criteria [8] and who had the first episode of their illness for one year or more preceding the outpatient follow up visits. The included adolescents were eighteen years of age and below as at the time of outpatient follow-up. The diagnoses of bipolar disorder in these adolescents were made independently by at least two trained psychiatrists based on criteria specified in DSM-IV. The co-morbid diagnoses in these adolescents were also made by independent assessment of at least two trained psychiatrists and the diagnoses made were based on criteria specified in DSM-IV

### Ethical consideration

Permission for the study was obtained from the Institutional Review Board of FNHE, Nigeria. The nature of the study was explained to the participants and consent was obtained from the adolescents and their caregivers.

### Instruments

#### Socio-demographic questionnaire

Socio-demographic questionnaire was designed to obtain information about gender, age, age at onset of illness, number of hospital admissions in the past year during the period of follow up, marital status of the parents among others.

### Procedure

Socio-demographic questionnaire was used to obtain demographic information from the participated adolescents. Co-morbid disorders were assessed through clinical interview, physical examination and laboratory investigations through the one year period of outpatient follow up. Clinical interview was also used to obtain history of co-morbid substance use, sexual risk behavior (defined as having unprotected sexual intercourse, intercourse with commercial sex workers, sexual intercourse with multiple partners without protection in the past year), level of religion activities which was rated as 'high' if the adolescent participated in worship/church activities for more than twice a week, 'moderate' if once to twice a week and 'low' if fortnightly or not at all. The adolescents were seen and interviewed together with their parents/guardians at various intervals on outpatient basis ranging between 4 to 10 outpatient visits within the period of one year depending on the individual adolescent. During the course of follow up on outpatient basis, some adolescents suffered relapsed symptoms of their illness and therefore were admitted as inpatients with continuation of the follow-up process on inpatient basis.

### Data analysis

Data were analyzed using Statistical Package for Social Sciences, (SPSS), version 15. Prevalence and pattern of co-morbid disorders were computed and qualitative inter-group data were compared with Chi-square test.

## Results

A total of forty six (46) adolescents were followed up. There were 29 (63.0%) males and 17 (37.0%) females. The age range of the adolescents was between 15 and 18 years, the mean age was 16.90 ± 1.07. The mean age at onset of bipolar disorder symptoms in the adolescents was 15.02 ± 1.02. The mean number of hospital admissions in the past year during follow up period among the adolescents was 2.85 ± 1.07. Twelve (26.1%), 16 (34.8%) and 18 (39.1%) of the adolescents were classified as having 'high', 'moderate' and 'low' level of religion activities respectively. Twenty eight (60.9%) and 18 (39.1%) of the parents of the adolescents were married and single parents respectively. Table 1 showed the socio-demographic variables and clinical parameters of the adolescents.

**Table 1:** Socio-demographic variables and clinical parameters of the adolescents

**Socio-demographic variables**	**N (%)**
**Gender**	
Male	29 (63.0)
Female	17 (37.0)
**Age (Years)**	
15	6 (13.0)
16	11 (23.9)
17	12 (26.1)
18	17 (37.0)
**Marital Status of the Parents**	
Married	28 (60.9)
Divorced/Separated/Widowed	18 (39.1)
**Co-morbid disorders**	
Present	22 (47.8)
Absent	24 (52.2)
**Level of Religion Activities**	
High	12 (26.1)
Moderate	16 (34.8)
Low	18 (39.1)
**History of Sexual Risk Behavior in the Past Year**	
Present	21 (45.7)
Absent	25 (54.3)
**Number of Hospital Admissions in the Past Year**	
1	6 (13.0)
2	12 (26.1)
3	11 (23.9)
4	17 (37.0)

### Prevalence and pattern of co-morbid disorders over a one year period

Twenty two (47.8%) of the adolescents had one or more co-morbid diagnoses. One (2.2%) had a co-morbid diagnosis of Human Immune-deficiency Virus (HIV) infection, a diagnosis that was made following presentation of clinical symptoms that led to the suspicion and subsequent laboratory confirmation. Four of the adolescents (8.7%) had co-morbid diagnosis of alcohol use disorders, 11 (23.9%) had co-morbid diagnosis of cannabis use disorders. Six (13.0%) of the adolescents had co-morbid diagnosis of conduct disorder with or without other psychoactive substances use. Table 2 showed the prevalence and pattern of co-morbid disorders in these adolescents.

**Table 2:** Prevalence and pattern of co-morbid disorders over a one year period

**Co-morbid disorders**	**N (%)**
Human Immune-deficiency Virus (HIV) Infection	1 (2.2)
Alcohol Use Disorders	4 (8.7)
Cannabis Use Disorders	11 (23.9)
Conduct disorder with or without other Psychoactive Substances Use	6 (13.0)

Combined Prevalence of Co-morbid disorders	22 (47.8)

### Gender specific prevalence of psychological co-morbidity

Males were more likely to experience one or more psychological co-morbidity compared to the females and this difference was statistically significant (χ^2 ^= 19.22, df = 3, p = 0.000). The gender specific prevalence of psychological co-morbidity is as shown in Table 3.

**Table 3:** Gender specific prevalence of psychological co-morbidity

**Gender**	**Alcohol Use Disorders**	**Conduct Disorder ± Other Psychoactive Substance Use**	**Cannabis Use Disorder**	**No Co-morbidity**	**Total**
**Male**	0	6	11	12	29
**Female**	4	0	0	13	17

**Total**	4	6	11	25	46

### History of sexual risk behavior and the associated factors

Twenty one (45.7%) of the adolescents had positive history of sexual risk behavior in the past one year. Having a history of sexual risk behavior was significantly associated with presence of co-morbid disorders (χ^2 ^= 8.91, df = 1, p = 0.003). History of sexual risk behavior was also significantly associated with level of religion activities (χ^2 ^= 37.70, df = 2, p = 0.000), with 'moderate' to 'high' level of religion activities offering protective effect on exhibiting sexual risk behavior. History of sexual risk behavior was also significantly associated with marital status of the parents (χ^2 ^= 5.34, df = 1, p = 0.021), with adolescents of single parents more likely to have history of sexual risk behavior.

## Discussion

About forty eight percent of the adolescents had co-morbid psychiatric and medical disorders in addition to the bipolar disorder. The pattern of co-morbid disorders included cannabis use disorders, alcohol use disorders, conduct disorder with or without other psychoactive substances use, and HIV infection, which was a chance finding heralded by presentation of symptoms that arose clinical suspicion of the diagnosis in one of the adolescents. Therefore, substance use disorders accounted for the significant ratio of co-morbidity in these adolescents with bipolar disorder. Substance use disorders had also been noted to be a prominent problem in healthy adolescents in the general population in Nigeria. However, the findings among these adolescents with bipolar disorder revealed some salient differences compared to what obtained in the past general population studies [9,10]. For example, the prevalence of alcohol use disorders of 8.7% found in this study is significantly lower than that of 15.6% found in one study that assessed people of comparative age groups in high school in Nigeria [9] and that of about 14% for past year in another study that cut across different age groups of Nigerian population [10]. Another interesting finding is that while alcohol use problems tend to be more common among the males in these general population studies [9,10], the contrast was the finding in these adolescents with bipolar disorder. The prevalence of 23.9% Cannabis use disorder found among these adolescents significantly exceeded the figure of 3.7% documented among people of comparable age range and 0.4% documented in people of different age range in Nigeria general population respectively [9,10]. The high prevalence of Cannabis use disorder in adolescents with bipolar disorder in this environment need to be further explored to determine factor(s) that may be responsible for this finding.

History of sexual risk behavior was present in about forty six percent of the adolescents. Having a history of sexual risk behavior was significantly associated with presence of co-morbid psychiatric and medical diagnoses. Having a history of sexual risk behavior was also significantly associated with level of religion activities and marital status of the parents, with 'moderate' to 'high' level of religion activities and married status of the parents offering protective effect on exhibiting sexual risk behaviors. The prevalence of 45.7% history of sexual risk behavior found in adolescents with bipolar disorder in this study is significantly higher than the observation of prevalence rates of 25.0 to 29.3% documented among healthy adolescents of comparative age drawn from the general population in one study [11]. This suggests that the presence of diagnosis of bipolar disorder in adolescents in this environment may make them more prone to exhibiting sexual risk behavior and its attendant consequences.

Co-morbidity had been documented to characterized early onset bipolar disorder [1,3,5]. The prevalence of co-morbid disorders of forty eight percent found in this study is comparable to the rates of co-morbid disorders, especially of substance use disorders found in earlier studies carried out in developed countries of the world [3,12].

Manic symptoms and co-morbid substance use disorders in adolescent bipolar disorder predispose affected adolescent to impulsivity, impaired judgment and risk taking behavior. These attributes could make such adolescent prone to sexual risk behavior. About forty six percent of adolescents that participated in this study had history of sexual risk behavior and our finding is in line with that of Meade et al [7] who found closely related prevalence of sexual risk behavior in patients with bipolar disorder. Co-morbid psychoactive substance use disorders accounted for the highest prevalence of co-morbidity among adolescents with bipolar disorder studied and a significant association was found between presence co-morbidity and history of sexual risk behavior. This finding concurred with that of Meade et al [7] that found association between history of sexual risk behavior and co-morbid psychoactive substance use disorders in patients with bipolar disorder.

Sexual risk behavior in these adolescents predisposes them to risk of sexually transmitted diseases (STDs). Presence of HIV infection as a co-morbid condition was a chance finding in one of these adolescents with bipolar disorder, this was because they were not routinely screened for any STDs including HIV infection unless there are symptoms presentation that arose suspicion of the diagnosis as occurred in one of these adolescents. Co-morbidity of HIV infection and other STDs in adolescent with bipolar disorder may need further evaluation in future studies. Although, the prevalence of about two percent presence of HIV co-morbidity among these adolescents is significantly lower than the rate of five percent documented as community prevalence of HIV infection in Nigeria by the 2003 Sentinel Survey [13], it still remained a pointer to the need to pay attention to issue of STDs in adolescents with bipolar disorder. Presence of mania symptoms had been noted to predispose individuals with bipolar disorder to engage in sexual risk behaviors and thus in turn predispose them to contracting HIV infection and it had also been documented that primary HIV infection in an individual may present with classical symptoms suggestive of mania. Therefore, a vicious cycle effect between mania symptoms and HIV infection had been suggested [14,15].

The association of history of sexual risk behavior with marital status of the parents, with adolescents of single parents being more likely to have such history underscored the importance of family and social dynamics in adolescent onset bipolar disorder outcome [5]. The family and social dynamics of adolescents with bipolar disorder should be given a good attention when planning interventions for such adolescents.

Presence of co-morbid disorders, especially psychoactive substance use disorders and sexual risk behavior and its attendant complications in adolescent onset bipolar disorder is a pointer to poor outcome and could cause the affected adolescents significant impairment in functioning [16,17] and mortality from risk taking behaviors and suicidal acts [18,19].

### Limitations

This is a clinical descriptive study that did not employ a case-control approach, although the findings of this study had been compared to previous findings in studies that assessed adolescents drawn from the general population sample. The socio-economic status of the parents which constitutes one of the factors in family and social dynamics could not be assessed because of the peculiar socio-cultural difficulty in volunteering information on income level in this environment. Another limitation of this study was that most of the information gathered on co-morbidity was based on unstructured clinical interview of the adolescents and their parents rather than using standardized algorithms. However, this is not expected to influence significantly the findings of this study because the interviews were conducted by trained specialists in psychiatry.

## Conclusion

Adolescents with bipolar disorder who suffer co-morbid disorders, especially psychoactive substance use disorders and who exhibit impulsivity and sexual risk behavior may need to be given special attention when planning interventions for children and adolescents with bipolar disorder. This may help to improve long term outcome in this group of adolescents. To the best of our knowledge, there are no past studies that have examined issue of co-morbidity in adolescent onset bipolar disorder in this environment and therefore the present data would provide a template for future longitudinal studies among adolescents with bipolar disorder in this environment.

## Competing interests

The authors declare that they have no competing interests.

## Authors' contributions

All authors contributed to the conception of the study. MOB wrote the initial draft of the manuscript and all authors were involved in revising the manuscript. All authors read and approved the final draft of the manuscript.
